# Spatiotemporal characterisation and risk factor analysis of malaria outbreak in Cabo Verde in 2017

**DOI:** 10.1186/s41182-018-0127-4

**Published:** 2019-01-07

**Authors:** Adilson José DePina, Alex Jailson Barbosa Andrade, Abdoulaye Kane Dia, António Lima Moreira, Ullardina Domingos Furtado, Helga Baptista, Ousmane Faye, Ibrahima Seck, El Hadji Amadou Niang

**Affiliations:** 1 Ecole Doctorale des Sciences de la Vie, de la Santé et de l’Environnement (ED-SEV), Université Cheikh Anta Diop (UCAD) de Dakar, Dakar, Sénégal; 2 Programa de Pré-Eliminação do Paludismo, CCS-SIDA, Ministério da Saúde e da Segurança Social, Praia, Cape Verde; 3 Instituto Nacional de Gestão do Território, Praia, Cape Verde; 4 Laboratoire d’Ecologie Vectorielle et Parasitaire, Faculté des Sciences et Techniques, Université Cheikh Anta Diop (UCAD) de Dakar, Dakar, Sénégal; 5 Programa Nacional de Luta contra o Paludismo, Ministério da Saúde e da Segurança Social, Praia, Cape Verde; 6 Delegacia de Saúde da Praia, Praia, Cape Verde; 7 Institut de Santé et Développement, Université Cheikh Anta Diop (UCAD) de Dakar, Dakar, Sénégal; 8 Aix Marseille Univ, IRD, AP-HM, MEPHI, IHU-Méditerranée Infection, Marseille, France

**Keywords:** Malaria, Spatiotemporal characterisation, Cabo Verde

## Abstract

**Background:**

Cabo Verde is a country that has been in the pre-elimination stage of malaria since the year 2000. The country is still reporting cases, particularly in the capital of Praia, where more than 50% of the national population live. This study aims to examine the spatial and temporal epidemiological profile of malaria across the country during the 2017 outbreak and to analyse the risk factors, which may have influenced the trend in malaria cases.

**Methods:**

Longitudinal data collected from all malaria cases in Cabo Verde for the year 2017 were used in this study. The epidemiological characteristics of the cases were analysed. Local and spatial clusters of malaria from Praia were detected by applying the Cluster and Outlier Analysis (Anselin Local Moran’s *I*) to determine the spatial clustering pattern. We then used the Pearson correlation coefficient to analyse the relationship between malaria cases and meteorological variables to identify underlying drivers.

**Results:**

In 2017, 446 cases of malaria were reported in Cabo Verde with the peak of cases in October. These cases were primarily *Plasmodium falciparum* infections. Of these cases, 423 were indigenous infections recorded in Praia, while 23 were imported malaria cases from different African countries. One case of *P. vivax* infection was imported from Brazil. Spatial autocorrelation analysis revealed a cluster of high-high malaria cases in the centre of the city. Malaria case occurrence has a very weak correlation (*r* = 0.16) with breeding site location. Most of the cases (69.9%, *R*^2^ = 0.699) were explained by the local environmental condition, with temperature being the primary risk factor followed by relative humidity. A moderately positive relationship was noted with the total pluviometry, while wind speed had a strong negative influence on malaria infections.

**Conclusions:**

In Cabo Verde, malaria remains a serious public health issue, especially in Praia. The high number of cases recorded in 2017 demonstrates the fragility of the situation and the challenges to eliminating indigenous malaria cases and preventing imported cases. Mosquito breeding sites have been the main risk factor, while temperature and precipitation were positively associated with malaria infection. In light of this study, there is an urgent need to reinforce control strategies to achieve the elimination goal in the country.

## Introduction

Despite global control efforts, malaria remains a major public health issue worldwide. According to the last World Health Organization (WHO) malaria report, nearly half of the world’s population is at risk [1]. For instance, in 2016, malaria transmission was reported in 91 countries and territories, mainly on the African continent, in Southeast Asia and in the eastern Mediterranean region [1–3]. The global health community has reinforced efforts against the disease with a renewed malaria elimination goal. By the end of 2016, at least 44 countries reported fewer than 10,000 cases, while 21 countries, including Cabo Verde, were eligible for malaria pre-elimination by 2020 [4, 5].

Cabo Verde is an archipelago of 10 islands in the Atlantic Ocean, approximately 570 km from the western African coast. The population of the country was estimated to be 537,661 inhabitants [6]. The archipelago is classified as a lower middle-income country with a gross domestic product (GDP) per capita of 2998 USD [7]. In the late 1950s, malaria was endemic in the country with 5000 to 15,000 cases per year and more than 200 malaria-related deaths [8]. Early control efforts through indoor residual spraying (IRS) with dichlorodiphenyltrichloroethane (DDT) and larval source management using both chemical larvicides and larvivorous fish have achieved the elimination of malaria twice for the country. The disease was then re-introduced twice afterwards [8, 9].

Presently, malaria is unstable throughout the country, with the burden being disproportionately high among men [10]. The country is characterised by a low malaria incidence of fewer than 1 case per 1000 inhabitants per year, despite the recent outbreak in 2017. In a previous study, we showed that indigenous cases were restricted to the islands of Santiago (mainly in the capital city of Praia) and Boavista. Imported cases from the African continent were more widespread across the country [10].

Entomological and parasitological surveys have reported *Anopheles arabiensis*, a member of the *Anopheles gambiae* complex, as the sole malaria vector in the country, and *Plasmodium falciparum* was responsible for almost all the malaria cases recorded between 2010 and 2016 [8, 10]. The archipelago’s malaria control programme integrates rapid diagnostics and quick treatment of all confirmed cases, as well as preventive measures, including vector control with IRS and larval source management. All confirmed cases are systematically treated with at least 3 days of hospitalisation and a reactive control response, including epidemiological and entomological surveys and spraying around the index case [11, 12].

Since Cabo Verde is targeting malaria for elimination by 2020, it is critical to evaluate the malaria transmission pattern in the country, considering the environmental and climatic factors influencing the disease epidemiology, as well as its control [13]. Furthermore, the integration of Geographical Information System (GIS) tools to map malaria incidence over a given space with the power of spatial statistical methods offers a robust predictive tool for the control of malaria [14–16]. The maps generated with GIS tools provide a visual representation of the cases across areas of interest, thereby enabling for the identification and location of areas of highest risk, where actions need to be targeted [16–18]. These advancements will further inform evidence-based decisions for the best use of limited resources in a cost-effective manner [18–21].

Studies throughout the world have indicated changes in malaria incidence due to patterns of environmental conditions [18–21]. In Cabo Verde, no study has examined the relationship between malaria epidemics and climatic factors, leading to the interest to better understand the spatial and temporal characteristics of malaria in the country, especially for transmission hotspots, such as in Praia.

In this study, we analysed epidemiological and/or environmental factors of risk potentially associated with the recent malaria outbreak recorded in Cabo Verde. This study provides critical epidemiological information which could be used by Cabo Verde decision makers to better target control strategies for the country’s elimination goal.

## Methods

### Study area

Surrounded by the sea, Cabo Verde is an archipelagic country characterised by a moderate climate and stable temperatures with extreme aridity. February is the coldest month (< 20 °C), while August and September are the hottest and wettest months, respectively (with temperatures > 20 °C). The Cabo Verde islands are profoundly affected by the two-season nature of the intertropical convergence zone (ITCZ). The climate of the archipelago and precipitation levels is attributed to the ITCZ with an average annual rainfall of 197 mm [22].

**Fig. 1:**
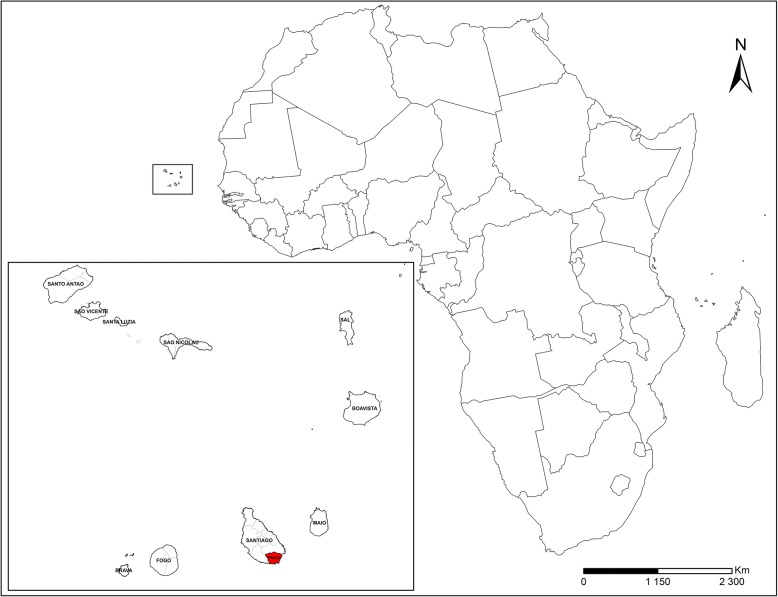
Location of the Cabo Verde islands

The study used the national data for malaria recorded in 2017. Spatial autocorrelation and cluster analysis were used for indigenous cases from Praia, the capital. Located on the largest island, Santiago, Praia, is described as a set of plateaus and respective surrounding valleys. Praia hosts 159,057 inhabitants representing approximately 29.2% of the national population in an area of 102.6 km^2^ and accounts for 43.2% of the overall national GDP (Fig. 1).

Praia has a desert climate (BWh, in the Köppen-Geiger climate classification) with a short rainy season and a dry season that runs from December to July and is characterised by constant winds (harmaton) from the Sahara Desert. The temperature is moderate, with the maximum rarely surpassing 32 °C and the minimum hardly ever being lower than 18 °C [23].

The municipality of Praia has recently recorded a high population growth rate of approximately 62% from 2000 to 2017. Almost 35.9% of the capital resident population come from other countries, with only 4.9% originating from the other islands of the archipelago. Seventy-two and 62% of the 44,079 households in Praia have access to electricity and piped water, respectively. Furthermore, 85.2% of the population have access to the sewage network. Approximately 28.1% of the population is classified as poor with a low level of education [24].

Praia is the principal malaria focus for Cabo Verde with 90% of indigenous cases in recent years from 2010 to 2016 [10]. For malaria control, the health delegation has a vector control team that goes to the field daily and performs the vector control activities, namely, identifying breeding sites, collecting identification information using a form, treating the larvae and surveying the municipality. In the study, all larval surveys were carried out at breeding sites, which were single pools of water with *Anopheles* present. The larval surveys had been done initially as a preventive activity, but with the notification of a malaria case, they were identified as part of the response to malaria cases. Data from January to December 2017 were used.

### Data collection

One of the greatest challenges for the control of malaria in low-transmission areas is its mapping, due to the concentration in hotspots and hot pops. In Cabo Verde, malaria notification is mandatory as part of the elimination programme. Therefore, all new cases are immediately reported and aggregated weekly in order to detect potential outbreaks immediately and to react in a timely manner. The individual notification form included personal information, residential address and supplementary information, which enabled the classification of each case as indigenous or imported. The data used in this study were retrieved from the National Malaria Control Programme (NMCP) and the National Surveillance Service, both operated under the authority of the Ministry of the Health (MoH) and Social Security. Individual data for each malaria case recorded from January to December 2017 were crosschecked and curated for completeness of the recorded information on the disease.

In order to infer the potential role of climatic factors, risk and transmission, meteorological data, which included monthly mean temperature, relative humidity, wind speed and rainfall, were obtained from the National Institute of Meteorology and Geophysics of Cabo Verde.

### Data analysis

#### Epidemiological analysis

The incidence of malaria by sex and age was estimated as the total number of confirmed cases in each group per 1000 inhabitants and was mapped by area for different regions of Praia using a choropleth map.

### Analysis of spatial autocorrelation and spatial distribution of malaria cases

To analyse the patterns of malaria and the spatial distribution of cases in Praia, a spatial autocorrelation analysis was performed using the Cluster and Outlier Analysis (Anselin Local Moran’s *I*) to identify statistically significant access points, cold points and spatial outliers and to estimate the spatial pattern of malaria cases [25]. This statistical test evaluates adjacent positions. A strong positive spatial autocorrelation is determined when the surrounding incidence of disease over the entire study area has analogous values. If the surrounding values are very different, the statistics point out a strong negative spatial autocorrelation. The value of Moran’s *I* Index varies between − 1 and 1. The *Z* value estimates whether the observed clustering/dispersing is statistically significant [26, 27]. Local Moran’s *I* Statistics was defined as follows:

$$ {I}_i\ (d)=\left({x}_i-\overline{x}\right)\ {\sum}_{j=1}^n\ {w}_{ij}(d)\left(x-\overline{x}\right),{}_{j\ne 1} $$

where $\overline{x}$was the annual incidence of *P. falciparum* cases in the area, *x*_*i*_ and *x*_*j*_ were the annual incidence of *P. falciparum* cases at district *i* and *j*, respectively, and *w*_*ij*_ was the spatial weight matrix based on the defined distance lags between area *i* and area *j*, where *w*_*ij*_(*d*) = 1 when neighbourhoods *i* and *j* were adjacent and 0 when *i* and *j* were not adjacent.

The Cluster-Outlier (CO) type field differentiates between a statistically significant (*p* < 0.01) cluster of high values (high–high), a cluster of low values (low–low), an outlier in which a high value is surrounded primarily by low values (high–low), and an outlier in which a low value is surrounded primarily by high values (low–high). A positive value for ‘*I*’ indicates that the adjacent districts are bounded by *P. falciparum* incidences with similar values. Such a feature is a cluster. A negative value for *I* indicates that the adjacent area is delimited by *P. falciparum* incidences with different values. Such a feature is an outlier. The Local Moran’s *I* can only be inferred with an estimated *Z*-score or *p* value. A 99% level of significance (*p* < 0.01) was used to designate important clusters of local autocorrelations [26, 27].

The patterns of malaria and spatially distributed cases were analysed for incidence (cases number/1000 population) [25, 26]. Statistically significant access points, cold points and spatial outliers were identified using statistics based on the inverse-distance relationship in which neighbours had a greater influence on calculations for a destination than distant locations and where distance was measured as a straight line between the two using Euclidean distance. The expected and observed index values were compared and the spatial correlation variance of malaria cases was determined using the *Z*-score. The *p* values were calculated to assess the significant differences of scores, and the pattern of spatial distribution was mapped to visualise the clusters of hotspots and cold areas.

### Analysis of risk factors

Potential risk factors implicated in the increase of malaria cases during the 2017 outbreak were assessed through a linear relationship analysis between the number of malaria cases and the presence of vector breeding sites using a multiple regression and a Pearson correlation. A temporal cluster analysis was used to detect which climatic risk factors most correlated with malaria cases throughout the year by integrating explicative variables such as the average temperature (°C), mean relative humidity (%), average wind speed (m/s), and total rainfall (mm) in the model.

## Results

### Epidemiological characteristics

A total of 446 cases of malaria were reported in Cabo Verde in 2017. Among these cases, 423 (94.8%) were indigenous cases from Praia, the capital of the country. The other 23 (5.2%) cases were imported, with infections being acquired outside of the country. During the same period, two malaria-related deaths were recorded, one in São Vicente and the other in Praia. Furthermore, 17 patients experienced two episodes of relapse while one patient experienced three episodes of relapse during the study period (Table 1).

**Table 1:** Number of malaria cases in Cabo Verde in 2017

	No. of cases	Percentage (%)
Indigenous	423	94.8
Imported	23	5.2
TOTAL	446	100
Death	2	0.4
Relapsed 2×	17	3.8
Relapsed 3×	1	0.2

Based on the WHO case classifications, which define imported cases as infections acquired outside of the country and indigenous cases as infections contracted locally with no evidence of importation, all the cases reported during the outbreak were correctly classified [4]. Praia, the capital city, recorded 423 cases (96.6%) followed by São Vicente with 7 cases (1.6%). All the other six municipalities had only one case each (0.2%) (Table 2). All of the indigenous and all the imported cases from African countries were identified as *P. falciparum* infections, while the single imported case from Brazil was a *P. vivax* infection. Notably, analysis of the patient travel histories showed that all the indigenous cases reported by the other municipalities originated from the capital city, Praia.

**Table 2:** Number of malaria cases in Cabo Verde by municipality and origin

Municipalities	Indigenous	Imported	Percentage (%)
Praia	423	13	97.8
São Vicente	0	7	1.6
Santa Catarina	0	1	0.2
Porto Novo	0	1	0.2
Sal	0	1	0.2
Total	423	23	100

### Indigenous cases

Further analysis of the indigenous cases recorded in Praia revealed that men were the most affected group (69.3%, *n* = 293). With a total incidence of 2.7/1000, the disease incidence was significantly higher among males than in females (3.7 and 1.7/1000, respectively). Although all age groups were affected by the outbreak (Table 3), young adults of 20–24 years of age were at highest risk, accounting for 13.7% (*n* = 58) of the total cases. The incidence was higher for the 50–54 age group and lower for the 0–4 age group, being 6.3 and 0.8/1000, respectively.

**Table 3:** Distribution of the malaria indigenous cases by sex and age in Praia in 2017

	Population	No. of malaria infections	Percentage (%)	Incidence (/1000)
Sex				
M	78,709	293	69.3	3.7
F	80,318	130	30.7	1.7
Age				
0–4	17,165	13	3.1	0.8
5–9	16,378	22	5.2	1.3
10–14	16,283	33	7.8	2.0
15–19	17,717	28	6.6	1.6
20–24	18,739	58	13.7	3.1
25–29	16,573	50	11.8	3.0
30–34	13,201	36	8.5	2.7
35–39	9560	37	8.7	3.9
40–44	8926	45	10.6	5.0
45–49	7813	20	4.7	2.6
50–54	5729	36	8.5	6.3
55–59	3553	20	4.7	5.6
60–64	1619	6	1.4	3.7
65+	5803	12	2.8	2.1
NA		7	1.7	
Total	159,057	423	100	*2.7*

*NA* data not available

**Fig. 2:**
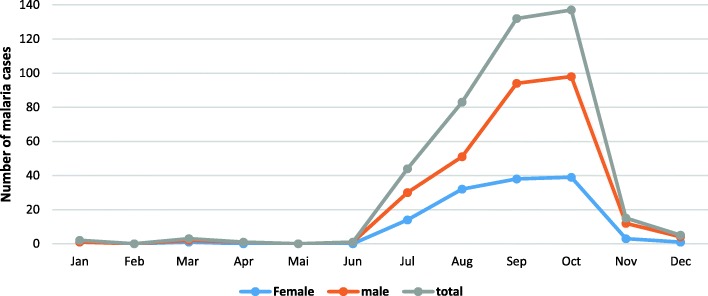
Temporal variation of malaria among males and females in Cabo Verde in 2017

Further analysis of the locally acquired cases revealed a temporal variation for malaria infection in Praia, mostly marked by a seasonal pattern. Indeed, only a small number of cases (five males and two female cases) were reported during the first semester of the year (January–June). Most of the cases were acquired between July and October, ranging from 44 to 147 cases. Disease incidence decreased towards the end of the year with 15 cases in November and 5 in December (Fig. 2).

### Imported cases

Investigation into the source of the imported malaria cases revealed that they originated mainly from the African continent, mostly from the Portuguese speaking countries of Angola and Guinea Bissau (21.7% each). The non-Portuguese speaking countries implicated were Senegal, Nigeria, and Guinea Conakry with five imported cases each. The remaining cases consisted of four *P. falciparum* infections from Benin, Ivory Coast, Burkina Faso and Ghana (one case each), and the single *P. vivax* infection imported from Brazil (Table 4).

**Table 4:** Origin of imported malaria cases in 2017

Imported	No. of cases	Percentage (%)
Angola	5	21.7
Guinea Bissau	5	21.7
Senegal	2	8.7
Guinea Conakry	2	8.7
Nigeria	2	8.7
NA	2	8.7
Benin	1	4.3
Ivory Coast	1	4.3
Burkina Faso	1	4.3
Ghana	1	4.3
Brazil	1	4.3
Total	23	100

*NA* data not available

### Spatial and temporal analysis of malaria case distribution in Cabo Verde in 2017

**Fig. 3:**
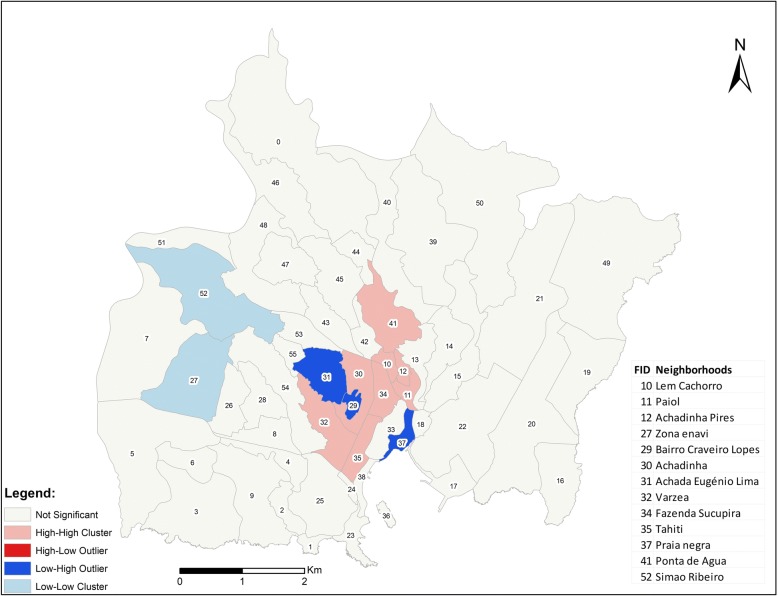
Spatial autocorrelation of malaria cases in Praia in 2017

The spatial analysis indicates a positive spatial autocorrelation and clustering (Moran’s *I* value = 0.22; *Z*-score = 4.15; *p* < 0.05) of malaria cases with three main patterns (Fig. 3).

**Fig. 4:**
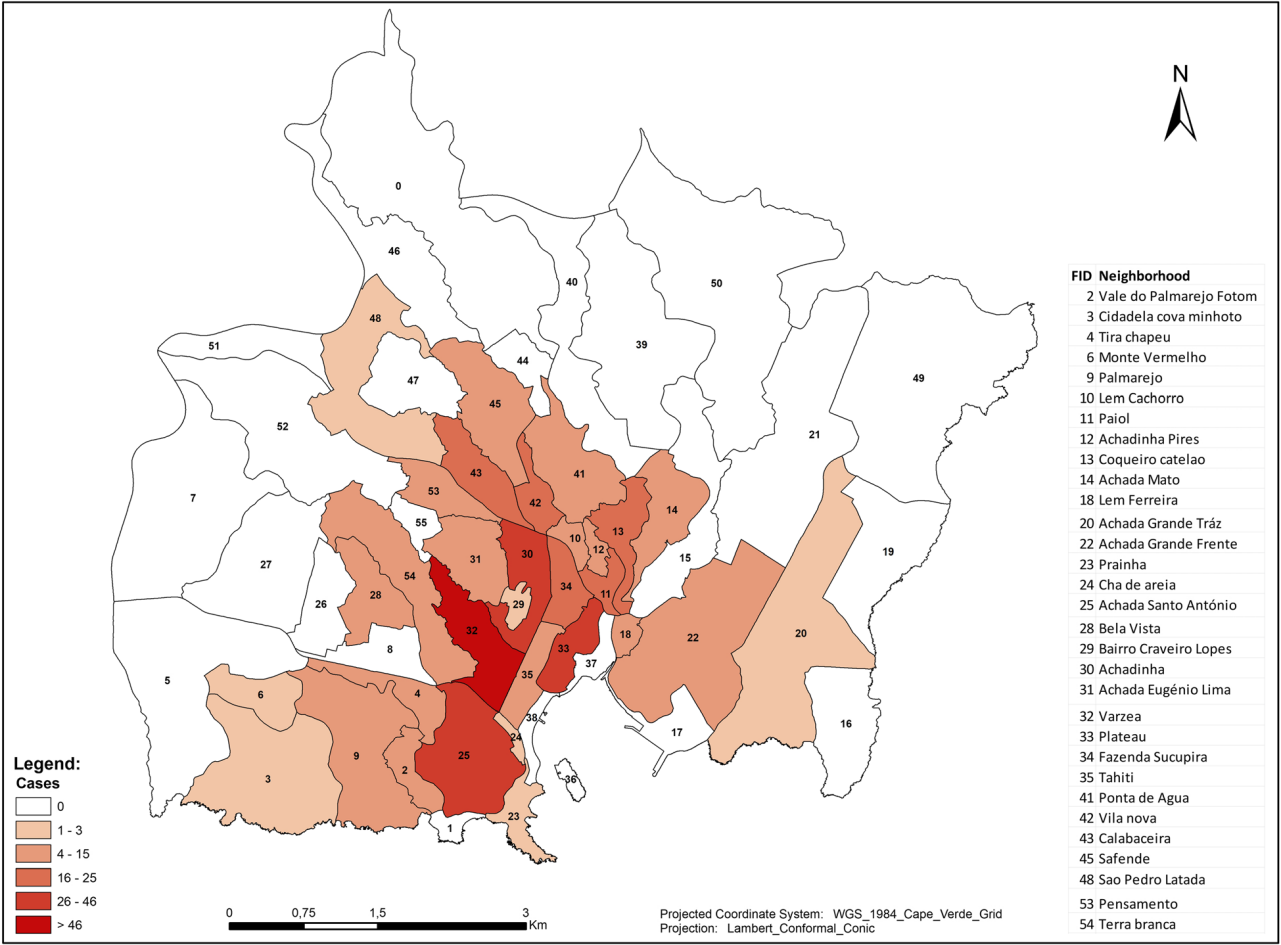
Cases of malaria in Praia, Cabo Verde, 2017

Notably, the spatial distribution of cases revealed that in Praia, the highest incidence of malaria was concentrated in the centre of the city, where a cluster of high-to-high incidence was identified within the neighbourhoods of Varzea, Fazenda/Sucupira, Tahiti, Lem Cachorro, Paiol, Achadinha Pires, Achadinha, and Ponta de Agua. In the western part of the city, the Simão Ribeiro and Envi Zone concentrated a cluster of low incidence. The rest of the area in Praia did not display any significant pattern (Figs. 3 and 4).

### Ecological and environmental risk factors associated with Malaria in Praia in 2017

Regression analysis allows for the modelling, examining and exploring of spatial relationships and helps to explain the factors behind the spatial patterns observed. This analysis can show why cases of malaria are higher in one neighbourhood than another, or which factors justify these cases of malaria in the modelling of spatial relationships.

**Fig. 5:**
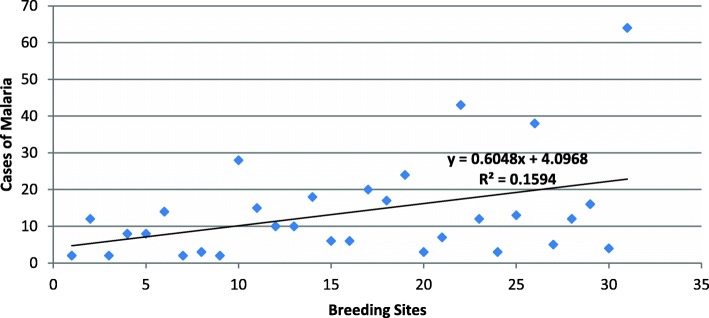
Association between positive breeding sites and cases of malaria in Praia, Cabo Verde, 2017

**Fig. 6:**
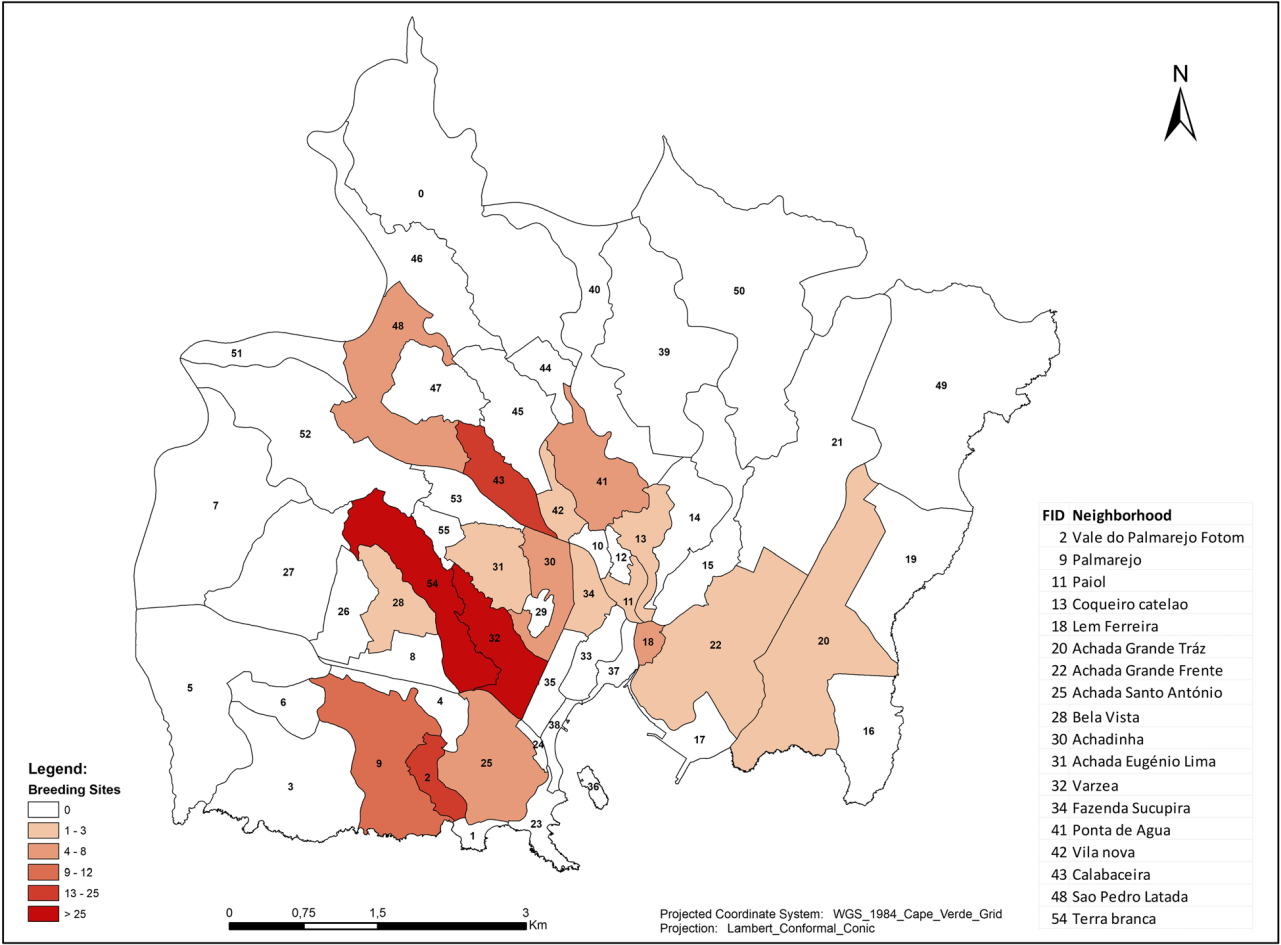
Variation of breeding sites by area in Praia, Cabo Verde, 2017

Figure 5 shows the association between the number of malaria cases in Praia in 2017 and the total number of positive breeding sites identified in neighbourhoods. We found a moderate positive linear correlation between malaria case occurrence and the breeding sites for the main malaria vector, *An. gambiae* s.l (Fig. 6).

**Fig. 7:**
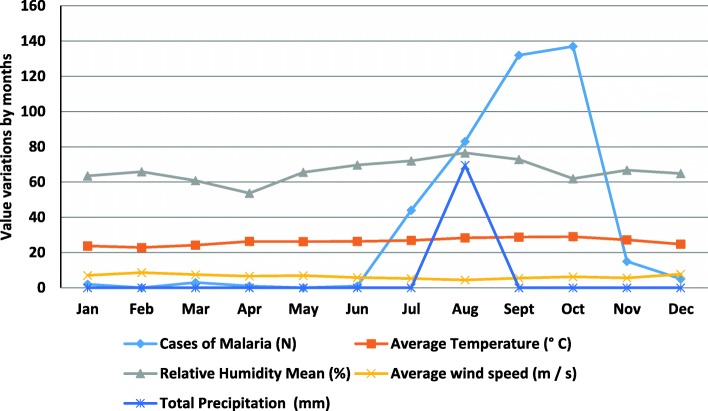
Monthly number of malaria cases and meteorological variables, Cabo Verde, 2017. Source: PNLP, 2018; INMG, 2018

The multiple linear regression analysis performed to assess the potential relationship between malaria cases and the meteorological variables, such as the mean temperature (°C), mean relative humidity (%), average wind speed (m/s) and total rainfall (mm), has shown that the 69.9% variation in malaria cases during the year for Praia is explained by the model with a highly positive linear correlation (*r* = 0.84). The mean temperature shows a strong relationship with malaria occurrence (*r* = 0.79) in Praia. Both the mean relative humidity (*r* = 0.39) and the total rainfall (*r* = 0.28) were moderately associated with the risk of malaria infection. Conversely, the average wind speed was negatively correlated to malaria (*r* = − 0.54). Figure 7 shows this correlation during the year.

The graph shown above illustrates the evolution of malaria cases and environmental data. The number of malaria cases increased after the first precipitation in July. Although there was no more rain in the following months, it seems that the precipitation in July was sufficient to increase the number of cases in the following months with a peak in October and a decrease in November and December. The data show a plateau of malaria cases just as the rains end.

**Fig. 8:**
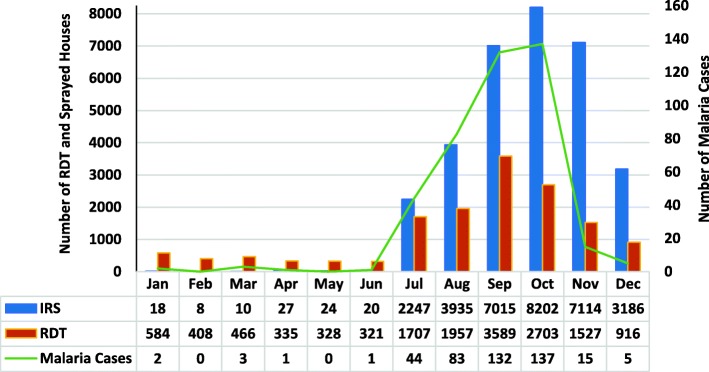
Number of rapid diagnostic test (RDT) and indoor residual sprayed (IRS) houses in Praia, Cabo Verde, during malaria outbreak 2017

After each case was notified by the health structure (hospital or health centre), a reactive response was initiated by the health delegation, and a team, including the epidemiological and entomological survey, allowed for a good classification of the cases. The reactive research into malaria after the index case, including the use of the rapid diagnostic test in the family and neighbour’s houses, the IRS, community mobilisation actions and other activities, the capacity of a good technician to manage cases, clinical and lab diagnosis, may all contribute to the reduction of cases and low mortality. Figure 8 shows the level of responsiveness following the number of cases reported based on the level of IRS and RDT.

## Discussion

### Epidemiology of malaria in Cabo Verde

This study investigated the epidemiological and spatiotemporal outbreak characteristics and risk factors of malaria for Praia, Cabo Verde, in 2017, as the country is in the pre-elimination stage [8]. Malaria elimination in Cabo Verde, which is set to be achieved by 2020, is significant for the health and economic development of Cabo Verde [5].

Studies show that progress in shrinking the malaria map with a focus on vector control, parasite incidence and other interventions, can maximise the impact on the control and elimination of the disease [13–18, 28]. In Cabo Verde, the mapping of malaria data remains a challenge. In recent years, the National Malaria Control Programme (NMCP) in partnership with national and international institutions has taken the first steps in this direction. With the outbreak of malaria in 2017, data on case location and breeding site identification began to be collected.

The indigenous cases in 2017 were centred in Praia, and a few imported cases were registered in other municipalities. There was a substantial increase in cases compared to records from recent years [10]. Praia continues to be a focus for malaria in the country, with the epidemiological and ecological factors necessary for malaria transmission [29], and for the last 2 years (2016–2017), all indigenous cases originated there [10, 27].

The results demonstrate that malaria transmission varies due to gender and age in the country. The high incidence rate observed in males in comparison with females might be due to different exposure rates or other behavioural risk factors. Similar studies have demonstrated the disproportionate burden by gender, with males being more affected, probably due to greater mobility and work-related activity in fields and risky areas [30, 31]. There are no studies on the profile of malaria patients in Cabo Verde, but the data on the notification forms from the last years indicate that men are more affected, particularly those who work as security, guards, the homeless and construction or agricultural workers. This finding is in contrast to those obtained in other countries in the African region, such as Mauritania, Senegal and Central Vietnam, where malaria affects mostly women and children under 5 years of age [32–34].

In Africa, malaria normally affects all age groups of the population in areas with unstable malaria transmission [33, 35–38]. In this study, the group aged 20–24 and 25–29 years old had the highest number of cases (58 and 50 cases, respectively), and the highest incidence rate was in the group aged 50–54 years (6.4/1.000 habitants).

The reported number of imported cases in 2017 was similar to that in recent years [10]. This finding is similar to that obtained in other countries in Africa, such as Reunion Island, where a considerable number of imported cases are from African countries [39], or Sri Lanka [40], a country in the process of malaria elimination with cases from South Asia, India and considerable numbers from Africa. However, the number of indigenous cases was more than 18 times higher than for imported cases.

The illness most frequently diagnosed in travellers all over the world is malaria [41], and Cabo Verde continues to receive cases of malaria from other countries, especially from the Lusophone world (Angola and Guinea Bissau) and from the West Africa region. This continuous history of imported cases presents a challenge for the country that in the context of elimination, Cabo Verde needs to have an excellent surveillance and response service, including information systems to allow for the identification, tracking, classification and response for all cases. Imported cases need to be followed and shown not to generate secondary cases, which are transmitted locally (introduced cases) [28].

The seasonality of cases in 2017 for Cabo Verde is similar to the previous years (2010–2016) [10] and to some others Sahelian countries [42, 43]. In these areas with seasonal malaria transmission, the incidence rate is presumed to be near zero in the dry season. During the first semester of the year (January–June), only 11 cases were reported in the country, with 7 of these being indigenous, representing only 2.5% of the total cases. All other cases were in the second semester of the year (July–December), the rainy period of the country, when the weather is especially hot and humid. The peak period for malaria cases in Cabo Verde differs from other countries in the elimination stage, such as Swaziland where there is a high prevalence in December to February [44].

The good organisation of health services in case management has made it possible to provide the best care for the hospitalised and treated cases, as well as for the response to cases, including the availability of RDT and IRS. This management has resulted in only two deaths, despite the high number of cases (0.2% mortality rate). One of the fatal cases was an imported case from Sao Vicente, and the other was a homeless case from Praia.

The high incidence of malaria is related to the complex geography and environmental conditions, such as the weather and climate [44, 45]. The hot and humid conditions are appropriate for breeding *Anopheles* [46], and the epidemiological characteristics of malaria in Cabo Verde showed an obvious seasonal pattern and demographic distribution that could be correlated with the high density and the capacity of the vectors to transmit the disease. The outbreak season for malaria in the country is during the summer, as shown in Fig. 2. Malaria incidence increased rapidly from July to October and decreased in November.

The areas with higher numbers of cases were identified as having high numbers of positive breeding sites. The results show a moderately positive linear correlation between the malaria cases in Praia and positive breeding sites (Fig. 5). This means that other factors had a significant contribution to the outbreaks of malaria in Praia. The results of the cluster map showed a high-high cluster of malaria cases in the centre of the city, encompassing the neighbourhoods of Várzea, Chã de Areia, Fazenda, Achadinha, Paiol and Lém Ferreira, during the outbreak. As mentioned before, several factors could explain this high-high cluster, as environmental, health and economic conditions have been found in studies conducted from others regions [44, 47, 48].

Environmental conditions and climate variables play an important role in the dynamics, distribution, and transmission of malaria and other vector-borne diseases. Generally, annual cumulative rainfall and annual average temperature were positively associated with the malaria incidence rate [49, 50]. Rainfall is critical in providing a suitable habitat and consequently has a significant impact on the survival rate of *Anopheles* mosquitoes. In 2017, the presence of breeding sites had a very weak correlation with the incidence of cases, which means that other determinants had a major impact on the malaria cases. Breeding sites as a result of low rainfall included the presence of permanent water troughs, agricultural areas, wells and other permanent ponds in the floodplain and surrounding areas, were principally identified during the malaria responses in Praia (Fig. 7). The fact that the early cases occurred in the Tahiti/Ponta Belém zone, peripheral to the areas of the large Sucupira market, where there is a large influx of population due to informal commerce from endemic African countries, could explain the increase and expansion of cases to other neighbourhoods of Praia and other municipalities of the Santiago island.

Temperature is a key driver that affects the essential processes of mosquito biology and parasite life cycle [51]. Temperature determines transmission intensity, including mosquito development rate, biting rate and survival of the parasite within the mosquito [50, 52]. In this study, the average annual temperature shows a strong positive correlation with malaria cases in Praia. A study of malaria distribution in ten West African countries (Benin, Burkina Faso, Côte d’Ivoire, Gambia, Ghana, Liberia, Mali, Senegal, Sierra Leone and Togo) during the period of 1996–2006 had similar results, where the two most important climate factors were found to be the average annual temperature and the total annual precipitation [45]. Other recent studies had similar results [52, 53]. It is important to note that, in cases where temperature extremes set boundaries on vector distributions, climate change might alter the range (in altitude or latitude) of favourable environmental conditions for malaria vectors and parasites. We noted that the greatest effect of climate change on malaria is likely to be observed at a temperature equal to 25 °C, corresponding to the favourable conditions for disease transmission [52].

There are temporal variations in the incidence of malaria that may be related to variations in climate and other local environmental risk factors [54, 55]. In this study, most malaria occurs from July to November with an increase of cases in September and a peak in October, immediately after the rains in August.

In addition to the direct influence of temperature on malaria incidence based on the biology of vectors and parasites, the precipitation patterns could also have effects on malaria in Africa [54–57]. We can see it in Cabo Verde, despite higher rainfall values in the past, that the number of cases of malaria was lower [10]. In this study, the pluviometry (mm) had a moderately positive correlation with cases of malaria. Of the other two factors analysed, the relative humidity showed a strong positive correlation and the average wind speed had a strong negative correlation, since wind has an impact on mosquito dispersion.

Climate change has been related to a rise in approximately 6% of malaria cases during 2000 for middle-income countries [56–58]. Environmental changes such as rainfall and temperature have been associated with other diseases like non-cholera diarrhoea, [59] visceral leishmaniasis [60] and others diseases. People in tropical areas and with lower educational levels are the most vulnerable to the effect of these factors.

The expected increases in temperature, changes in precipitation patterns, and increased flooding and drainage are important issues in developing and archipelagic countries, like Cabo Verde [61–63]. The impact of these factors on water-borne and vector-borne diseases is of particular relevance in terms of policies and public health strategies, regarding the prevention of these diseases. Further studies considering multiple areas and biological models of mosquito development are needed to improve detection of temperature effects on malaria transmission and the relative contribution of these risk factors.

## Conclusions

This study was conducted to analyse the cases of malaria in Cabo Verde during the outbreak in 2017 in Praia. Men and people > 20 years old were more affected by the disease in the country. Imported cases were from the Lusophone world, and one case of *P. vivax* from Brazil was identified. The reporting of cases had the same epidemiological tendencies, which were recorded mainly between July and October. Cabo Verde has an excellent case management programme, which resulted in a reduction in the mortality rate.

Measures on the effect of environmental factors for malaria showed a strong positive correlation with temperature and relative humidity, a moderately positive correlation with the pluviometry and a strong negative correlation with wind speed. Findings from this study shall help policy and decision makers in preventive measures for malaria control and to achieve the malaria elimination goal for this country. More studies are recommended to complete these results and elaborate on surveillance plans and activities to adequately achieve malaria elimination in the country.

## Abbreviations

BWh: Hot desert climate
DDT: Dichlorodiphenyltrichloroethane
FID: Features identify
GDP: Gross domestic product
IRS: Indoor residual spraying
MoH: Ministry of Health
NMCP: National Malaria Control Programme
RDT: Rapid diagnostic test
WHO: World Health Organization
